# *WT1*, *NR0B1*, *NR5A1*, *LHX9*, *ZFP92*, *ZNF275*, *INSL3*, and *NRIP1* Genetic Variants in Patients with Premature Ovarian Insufficiency in a Mexican Cohort

**DOI:** 10.3390/genes13040611

**Published:** 2022-03-29

**Authors:** Luis Ramos

**Affiliations:** Department of Reproductive Biology, Instituto Nacional de Ciencias Médicas y Nutrición, Salvador Zubirán, Ciudad de México 14080, Mexico; luis.ramost@incmnsz.mx; Tel.: +55-5487-0900

**Keywords:** ovary, fertility, polygenic, synonymous SNVs, non-synonymous SNVs, non-coding SNVs

## Abstract

Premature ovarian insufficiency (POI) is one of the main causes of female premature infertility. POI is a genetically heterogeneous disorder with a complex molecular etiology; as such, the genetic causes remain unknown in the majority of patients. Therefore, this study aimed to identify mutations and characterize the associated molecular contribution of gonadogenesis-determinant genes to POI. Genomic assays, including PCR-SSCP and Sanger sequencing, followed by in silico analyses were used to investigate the underpinnings of ovarian deficiency in 11 women affected by POI. Large deletions and nucleotide insertions and duplications were excluded by PCR. Thirteen genetic variants were identified in the *WT1* (c.213G>T, c.609T>C, c.873A>G, c.1122G>A), *NR0B1* (c.353C>T, c.425G>A), *NR5A1* (c.437G>C, IVS4-20C>T), *LHX9* (IVS2-12G>C, IVS3+13C>T, c.741T>C), *ZNF275* (c.969C>T), and *NRIP1* (c.3403C>T) genes. Seven novel genetic variants and five unpublished substitutions were identified. No genetic aberrations were detected in the *ZFP92* and *INSL3* genes. Each variant was genotyped using PCR-SSCP in 100 POI-free subjects, and their allelic frequencies were similar to the patients. These analyses indicated that allelic variation in the *WT1*, *NR0B1*, *NR5A1*, *LHX9*, *ZFP92*, *ZNF275*, *INSL3*, and *NRIP1* genes may be a non-disease-causing change or may not contribute significantly to the genetics underlying POI disorders. Findings support the polygenic nature of this clinical disorder, with the SNVs identified representing only a probable contribution to the variability of the human genome.

## 1. Introduction

Ovarian longevity is physiologically critical for fertility and impacts reproductive aging in women. The loss of ovarian function in women, referred to as premature ovarian insufficiency (POI, OMIM #311360), is associated with oligomenorrhea/amenorrhea, sometimes with hypoplastic ovaries undetectable upon pelvic ultrasound, elevated gonadotropins (particularly FSH) in repeated blood tests (>4 weeks apart), and low sex steroid hormone levels (particularly estradiol) before age 40 [1,2,3] (https://www.eshre.eu/Guidelines-and-Legal/Guidelines/Management-of-premature-ovarian-insufficiency.aspx, accessed on 1 March 2022). POI is a severe reproductive disorder affecting approximately 1% of women of childbearing age worldwide, resulting in increased use of assisted reproduction techniques (ARTs) such as in vitro fertilization (IVF). POI not only interferes with a woman′s reproductive potential, but is also associated with reduced bone mineral density, an increased risk of cardiovascular disease, and earlier mortality [4,5].

The genetic and molecular etiologies of POI have been described in the context of chromosomal abnormalities or rare gene defects. Turner syndrome (45, X) is the most common cytogenetic cause of POI. Trisomy X (47, XXX) is an X chromosome aneuploidy presenting with POI characterized by elevated FSH levels. Other X chromosome aberrations include deletions/duplications and balanced/unbalanced X-autosome rearrangements [6,7,8,9]. Many regions on the X chromosome are critical for healthy ovarian development, and these also contain multiple genes (e.g., *DIAPH2*, *XPNPEP2*, *DACH2*) associated with POI. Specifically, the region on the long arm of the X chromosome from Xq13–Xq21 to Xq23–Xq27 is associated with the POI phenotype [10,11,12]. However, X-structural anomalies and X-autosome translocations have not been completely useful for relating to POI; therefore, additional heretofore unknown genetic factors are likely also involved in the etiology of this disease.

To date, more than 100 genes have been implicated in the loss of ovarian activity [8,13,14,15], and single-gene mutations have been associated with POI [16,17,18]. The genes involved in the pathogenesis of POI also affect development [19,20], DNA division and repair [21,22], follicle development and hormonal signaling [23,24], metabolism [25,26], and immune regulation [27,28]. The premutation of the fragile X mental retardation 1 (*FMR1*) gene is the most common genetic factor associated with POI. The *FMR1* allele is located on the X chromosome at q27.3 and contains a trinucleotide repeat sequence, (CGG)n, in its 5′ untranslated region. This gene contains 6–44 CGG repeats with AGG interspersions every 9 or 10 repeats, and having over 55 CGG repeats is associated with pathogenicity [29,30,31,32,33,34].

Previous reports demonstrate the scarcity of knowledge concerning the molecular etiology of this ovarian disorder and highlight the importance of identifying novel genetic candidates potentially linked to the pathogenesis of POI. The genetic and molecular mechanisms underlying this disease, which could inform the development of more effective treatments to preserve fertility, have not yet been fully elucidated. To gain further insight into the contribution of gonadogenesis-determinant genes to the pathogenesis of POI, the current study examined the frequency and downstream molecular implications of *WT1* (11p13), *NR0B1* (Xp21.2), *NR5A1* (11q13.1), *LHX9* (1q31.3), *ZFP92* (Xq28), *ZNF275* (Xq28), *INSL3* (19p13.11), and *NRIP1* (21q11.2-q21.1) mutations in patients with POI. I selected these eight genes for genetic analysis in POI patients because they have been associated with gonadogenesis (specifically involved in sex determination, and these are determinant factors of gonadal function in humans and model animals, such as *WT1*, *NR0B1*, and *NR5A1*), and in many cases, their biological functions (such as *ZFP92* and *ZNF275*) and clinical implications (such as *LHX9*, *INSL3*, and *NRIP1*) are unknown.

## 2. Materials and Methods

### 2.1. Patients and Participants

All of the female patients (*n* = 11) were under the age of 40 years and had a 46, XX karyotype, with a body mass index between 23 and 28, high levels of gonadotropins (FSH > 20 IU/L; LH > 30 IU/L), hypoestrogenism (<10 pg/mL), hypogonadism, and amenorrhea. The exclusion criteria included menopause caused by hysterectomy, bilateral ovariectomy, radiation or chemotherapy, and autoimmune diseases. The study included 100 unrelated, healthy female subjects of reproductive age (16–40 years) as controls, with positive fertility and without assisted reproductive therapy, who were recruited from the Department of Reproductive Biology, Instituto Nacional de Ciencias Médicas y Nutrición Salvador Zubirán (INCMNSZ). All subjects had Mexican ancestry, and all of them were screened for the *WT1* (NM_024426.6), *NR0B1* (NM_000475.5), *NR5A1* (NM_004959.5), *LHX9* (NM_020204.3), *ZFP92* (NM_001136273.2), *ZNF275* (NM_001080485.4), *INSL3* (NM_001265587.2), and *NRIP1* (NM_003489.4) genes. This study was approved by the Human Ethics Committee of the INCMNSZ (reference number: BRE-3594-21-24-1).

### 2.2. gDNA Extraction

Genomic DNA (gDNA) was isolated from 10 mL of peripheral blood leukocytes collected in EDTA (0.5 M, pH = 8). One volume of whole blood was diluted with 3.5 volumes of cold buffer (0.64 M sucrose, 0.01 M MgCl_2_, 2% Triton X-100, and 0.02 M Tris-base at pH = 7.6) and then homogenized by inversion at 4 °C for 10 min. After centrifugation (1000× *g*, 15 min) at 4 °C, the nuclear pellet was resuspended in 3 mL of cold solution (10 mM Tris-base, 400 mM NaCl, 2 mM Na_2_EDTA), 108 µL of 20% SDS, and 100 µL of proteinase K (5 mg/mL). The mixture was incubated for 2 h at 50 °C. Saturated NaCl (0.3 volumes) was added, and the mixture was centrifuged at 1000× *g* for 15 min. The gDNA was precipitated from the supernatant by adding two volumes of 100% ethanol and then gently resuspended in 500 µL of a 1 M Tris, 0.5 M EDTA solution. The purity (260/280 = 1.8–1.9) and concentration (300 ng/µL) of each gDNA sample were determined spectrophotometrically (Beckman DU 650, Fullerton, CA, USA) by the A260/A280 absorbance ratio. The gDNA samples were stored at −20 °C until further analysis.

### 2.3. Genetic Screening and Genotyping

Mutations were identified via PCR–single-strand conformation polymorphism (SSCP) analysis. All coding exons of the *WT1* (NM_024426.6; Region 11: 32409321–32457176), *NR0B1* (NM_000475.5; Region X: 30322323–30327715), *NR5A1* (NM_004959.5; Region 9: 127243516–127269709), *LHX9* (NM_020204.3; Region 1: 197881037–197904608), *ZFP92* (NM_001136273.2; Region X: 152683780–152687087), *ZNF275* (NM_001080485.4; Region X: 152599613–152625568), *INSL3* (NM_001265587.2; Region 19: 17927321–17932383), and *NRIP1* (NM_003489.4; Region 21: 16333556–16437321) genes, including their intron boundaries, were individually amplified from 300 ng/µL of gDNA via polymerase chain reaction (PCR) using (α-^32^P)-dCTP-specific activity (3000 Ci/mmol; PerkinElmer, Boston, MA, USA) and GoTaq Flexi DNA Polymerase, according to the supplier′s guidelines (Promega, Madison, WI, USA) in a final reaction of 20 µL. The PCR reactions contained 25 µM of dNTP and 20 µM of each sense and antisense oligonucleotide (Supplementary Tables S1–S8). For all exons, the MgCl_2_ concentrations and melting temperatures were calculated experimentally. The PCR conditions were: 1 cycle at 94 °C for 3 min; 30 cycles at 94 °C for 30 s, 57–65 °C for 30 s, and 72 °C for 30 s; and 1 final extension at 72 °C for 3 min. The (α-^32^P)-dCTP-PCR reactions were visually assessed on 1% agarose gels containing 0.5 μL/100 mL ethidium bromide run at 100 V for 1 h. A total of 77 agarose gels were required to analyze the eight candidate genes. Exonic amplifications were observed using a UV transilluminator (Molecular Imager Gel Doc XR System, BioRad Laboratories, Hercules, CA, USA). Amplicon size was determined by comparison to a 100 bp molecular weight marker.

To detect genetic variants or exonic mutations, each exon amplified via (α-^32^P)-dCTP-PCR was analyzed by electrophoresis in four denaturing polyacrylamide gels using SSCP. After each (α-^32^P)-dCTP-PCR amplification, 1 mL of (α-^32^P)-dCTP-PCR reaction was mixed with 14 mL of loading buffer (95% formamide, 20 mM EDTA, 0.05% bromophenol blue). The samples were denatured at 95 °C for 5 min and cooled for 5 min. One microliter of each sample was loaded onto four polyacrylamide gels. Two 8% polyacrylamide gels with and without glycerol (7 mL) were prepared using 14 mL of TBE 5X, 18.66 mL of acrylamide-*N*,*N*′-methylenebisacrylamide (29:1), 0.48 mL of 10% ammonium persulfate (APS), and 25 µL of tetramethylethylenediamine (TEMED) in a final volume of 70 mL with H_2_O, and two 5.4% polyacrylamide gels with and without glycerol (7 mL) were prepared using 14 mL of TBE 5X, 12.6 mL of acrylamide-*N*,*N*′-methylenebisacrylamide (29:1), 0.48 mL of 10% APS, and 24.5 µL of TEMED in a final volume of 70 mL with H_2_O. Electrophoresis was carried out at 200–250 volts for 18 h at room temperature, after which the polyacrylamide gels were dried for 1 h at 75 °C and exposed to Imaging Screen-K screens for 3 h. A total of 308 polyacrylamide gels were required to screen the eight candidate genes. The results were analyzed using the Personal Molecular Imager System (Bio-Rad Laboratories, Hercules, CA, USA). Exons with aberrant migration patterns on the SSCP gels were amplified by PCR without (α-^32^P)-dCTP and purified using electroelution (Spectra/Por 1 Dialysis Membrane, MWCO: 6–8 kD, Spectrum Laboratories, Inc, Rancho Dominguez, CA, USA) and Amicon Ultra-4, Ultracel-10 K centrifugal filter devices at 1500× *g* for 20 min (Merck Millipore Ltd., Carrigtwohill, Co., Cork, Ireland). The allelic and genotypic frequencies of 100 unrelated, healthy subjects were determined using PCR-SSCP assays.

### 2.4. Variant Sequencing

Sanger sequencing was carried out to identify the genetic variants found via PCR-SSCP analysis from the patients with POI. The purified coding exons with aberrant genetic profiles were sequenced with the BigDye Terminator v3.1 Cycle Sequencing kit (Applied Biosystems, Austin, TX, USA) in a 10 µL reaction containing 10 ng/µL, 1 µL of sense or antisense oligonucleotide (20 µM), 1 µL of 5X sequencing buffer, and 2 µL of sequencing RR-100. Thermal cycling was carried out in a Veriti 96-well Thermal Cycler (Applied Biosystems, Austin, TX, USA) for 1 min at 96 °C followed by 35 cycles of 96 °C for 10 s, 50 °C for 5 s, and 60 °C for 4 min. The sequencing reactions were purified with 45 µL of SAM buffer and 10 µL of XTerminator solution according to the manufacturer′s protocol (BigDye XTerminator Purification Kit; Applied Biosystems, Austin, TX, USA). The samples were then vortexed for 30 min (2000 rpm; BV1000 Vortex Mixer, Edison, NJ, USA) and centrifuged at 1000× *g* for 2 min at room temperature. The sequencing reactions were analyzed using the ABI Prism 310 Genetic Analyzer (Applied Biosystems, Foster City, CA, USA) and then subjected to capillary electrophoresis using the run module KB_310POP6_BDTv3_36Rapid (temperature: 50 °C; injection voltage: 15 kV; injection time: 15 s; 5 to 8 μA). The dideoxy sequencing data were analyzed using Chromas software.

### 2.5. Genetic Variant Analysis

Four prediction programs for disease-associated variants in human genes (MutationTaster2 (https://www.genecascade.org/MutationTaster2021/#transcript, accessed on 1 December 2021), PolyPhen-2 (http://genetics.bwh.harvard.edu/pph2/, accessed on 1 December 2021), PROVEAN (http://provean.jcvi.org/seq_submit.php, accessed on 1 December 2021), and VarSite (https://www.ebi.ac.uk/thornton-srv/databases/VarSite, accessed on 1 December 2021) were used to display the mutational/functional impact of the genetic variants. The reference numbers (https://www.uniprot.org/, accessed on 1 December 2021) and amino acid sequences (https://www.ncbi.nlm.nih.gov/gene/, accessed on 1 December 2021) were obtained for WT1 (P19544; NP_077744.4), NR0B1 (P51843; NP_000466.2), NR5A1 (Q13285; NP_004950), LHX9 (Q9NQ69; NP_064589.2), ZNF275 (Q9NSD4; NP_001073954.3), and NRIP1 (P48552; NP_003480). Genetic variants were compared and assessed using the 1000 Genomes Project (https://www.ncbi.nlm.nih.gov/variation/tools/1000genomes/, accessed on 1 December 2021) and Genome Aggregation Database (https://gnomad.broadinstitute.org/, accessed on 1 December 2021). The genotypic and allelic data were analyzed using GenAIEx V 6.41 software. The Hardy–Weinberg (H-W) equilibrium of each variant was assessed using Pearson’s chi-square.

## 3. Results

### 3.1. Exonic Evaluation

**Figure 1:**
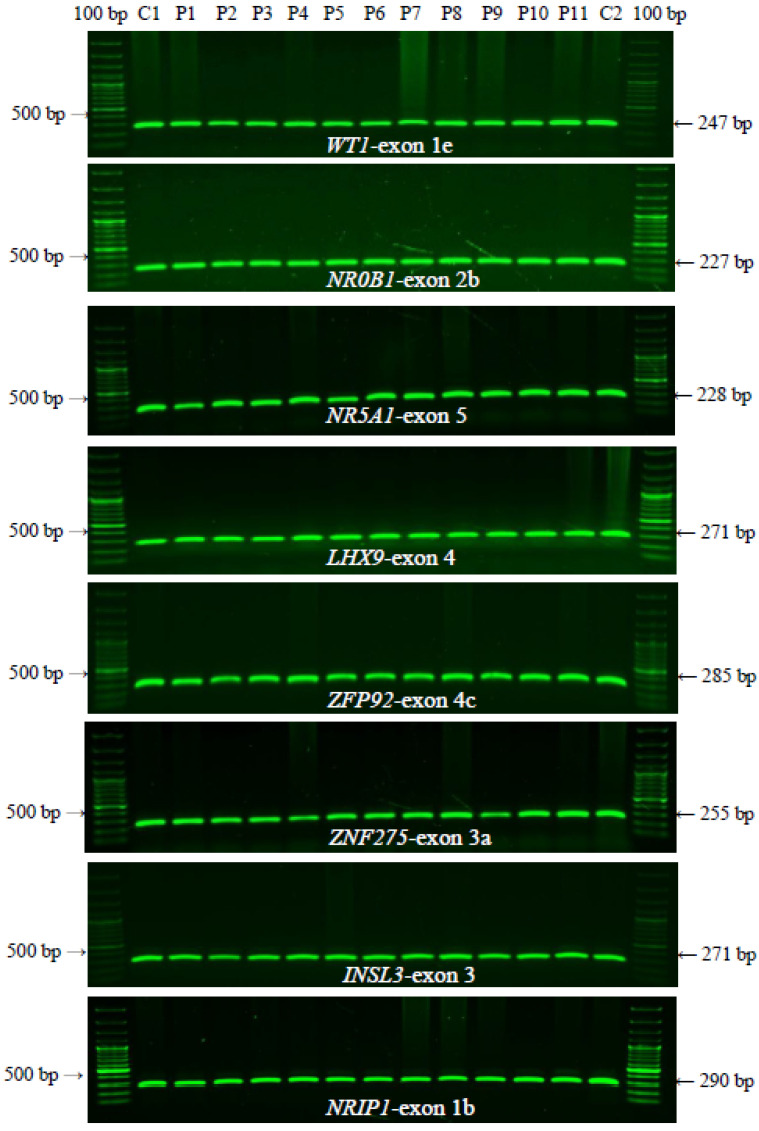
Representative banding patterns for the *WT1* (NM_024426.6), *NR0B1* (NM_000475.5), *NR5A1* (NM_004959.5), *LHX9* (NM_020204.3), *ZFP92* (NM_001136273.2), *ZNF275* (NM_001080485.4), *INSL3* (NM_001265587.2), and *NRIP1* (NM_003489.4) genes. All exons were amplified by (α-^32^P)-dCTP-PCR, and one representative exon is shown for each gene. The arrows indicate 500 bp, and 100 bp indicates the molecular weight marker (100–1500 bp). C1 and C2 are unrelated healthy subjects. P1–P11 are patients clinically classified with POI. The expected sizes for the amplified exons are shown on the right.

To gain insight into potentially important gonadogenesis-determinant genes and ovarian function associated with POI, genetic variants or exonic/coding mutations were investigated by PCR-SSCP-sequencing assays and pathogenicity prediction programs. Eight candidate genes (*WT1* (NM_024426.6), *NR0B1* (NM_000475.5), *NR5A1* (NM_004959.5), *LHX9* (NM_020204.3), *ZFP92* (NM_001136273.2), *ZNF275* (NM_001080485.4), *INSL3* (NM_001265587.2), and *NRIP1* (NM_003489.4)) were analyzed. Figure 1 contains a representative image of one exon from each gene to illustrate the specific exonic amplifications analyzed in the eight candidate genes identified in the patients with POI (P1–P11) and two healthy subjects (C1 and C2). The PCR amplicons exhibited an expected molecular size of 200–300 bp, similar to the healthy control subjects. Extensive insertions, deletions, and duplications were excluded in all of the exonic regions analyzed by PCR.

### 3.2. Mutation Screening

**Figure 2:**
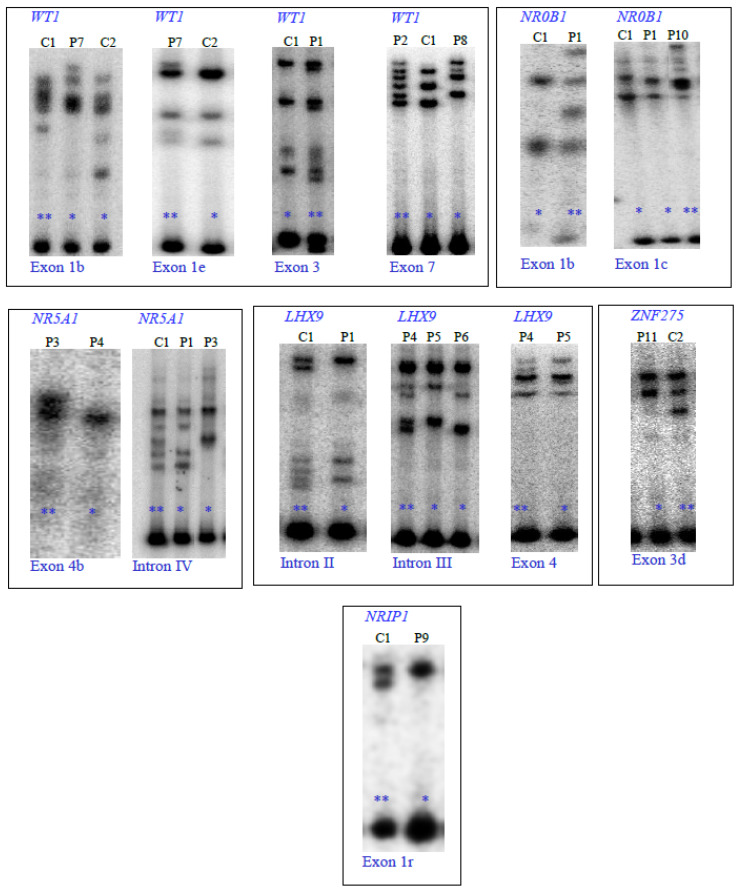
Genetic variants identified using (α-^32^P)-dCTP-PCR-SSCP analysis of the *WT1* (NM_024426.6), *NR0B1* (NM_000475.5), *NR5A1* (NM_004959.5), *LHX9* (NM_020204.3), *ZNF275* (NM_001080485.4), and *NRIP1* (NM_003489.4) genes from patients with POI (P) and healthy subjects (C). Different SSCP patterns are marked by asterisks. Heterozygous and homozygous subjects are indicated by two and one asterisks, respectively.

From the PCR-SSCP assays, I identified 13 genetic variants in six (*WT1*, *NR0B1*, *NR5A1*, *LHX9*, *ZNF275*, and *NRIP1*) of the eight candidate genes. Figure 2 illustrates the molecular identification of four genetic variants in the *WT1* gene (exon 1b: three different SSCP patterns, exon 1e: two different SSCP patterns, exon 3: two different SSCP patterns, and exon 7: three different SSCP patterns); two genetic variants in the *NR0B1* gene (exon 1b: two different SSCP patterns and 1c: three different SSCP patterns); two genetic variants in the *NR5A1* gene (exon 4b: two different SSCP patterns and intron IV: three different SSCP patterns); three genetic variants in the *LHX9* gene (intron II: two different SSCP patterns, intron III: three different SSCP patterns, and exon 4: two different SSCP patterns); one genetic variant in the *ZNF274* gene (exon 3: two different SSCP patterns); and one variant in the *NRIP1* gene (exon 1r: two different SSCP patterns); while no variations were detected in the *ZFP92* and *INSL3* genes.

### 3.3. Gene Variants

**Figure 3:**
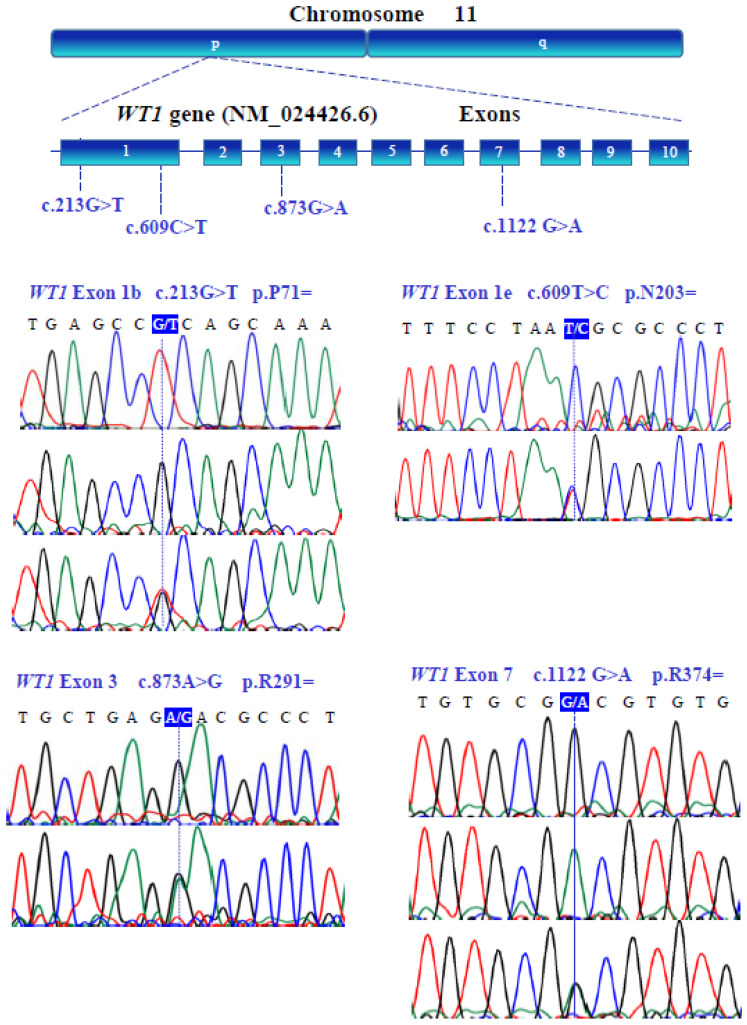
Schematic illustration of amplified exons from the human *WT1* (NM_024426.6) gene located on chromosome 11. Exons (1–10) are represented by blue boxes. Partial nucleotide sequences of the human *WT1* gene were obtained by bidirectional Sanger sequencing from patients with POI. The human genome sequence revealed that the *WT1* gene contains four synonymous polymorphisms.

**Figure 4:**
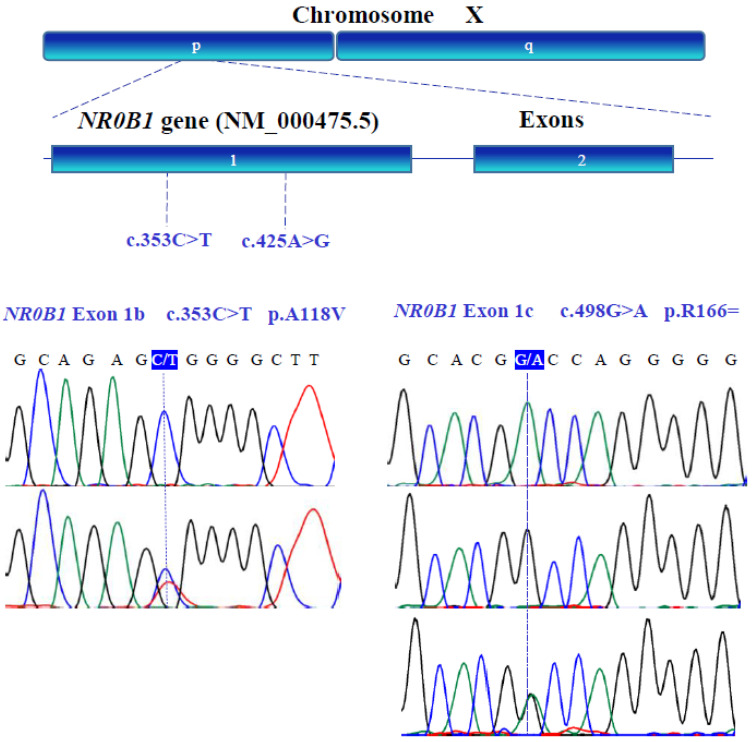
X-chromosome and exon representations of the human *NR0B1* (NM_000475.5) gene. Exons (1 and 2) were analyzed by capillary electrophoresis. Human *NR0B1* gene sequence variants were identified in exon 1 (blue box) from patients with POI. The presented fragment of the nucleic acid sequence of the *NR0B1* gene contains both the non-synonymous and synonymous variants.

**Figure 5:**
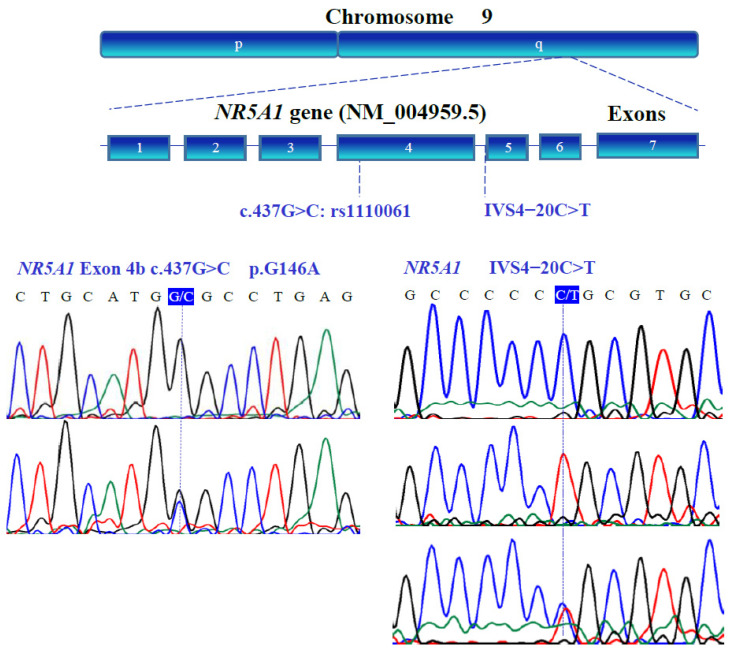
Partial nucleotide sequence of the human *NR5A1* (NM_004959.5) gene and a structural diagram of its location on chromosome 9. Genotypic variants in the coding/non-coding regions are located in exon 4 and intron 4 (blue boxes), respectively. Exons are indicated with blue boxes.

**Figure 6:**
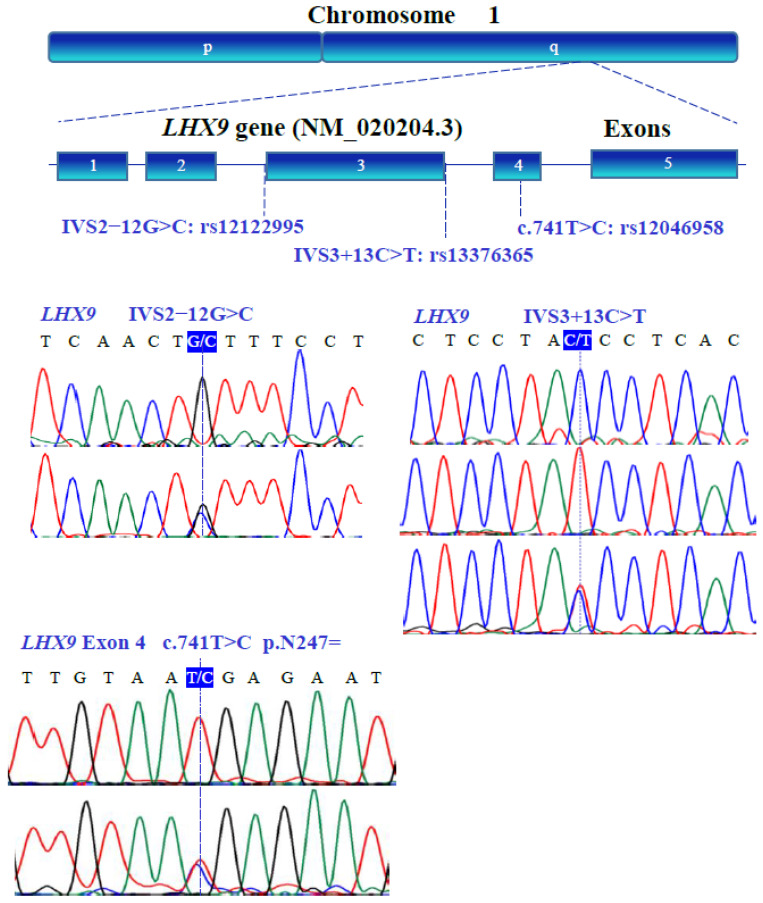
Allelic variants of the human *LHX9* (NM_020204.3) gene located on the long arm of chromosome 1. Five exons (blue boxes) were assessed by PCR-SSCP analysis and the coding/non-coding variants were identified by DNA sequencing. The locations of the substitutions are indicated above the electropherogram.

**Figure 7:**
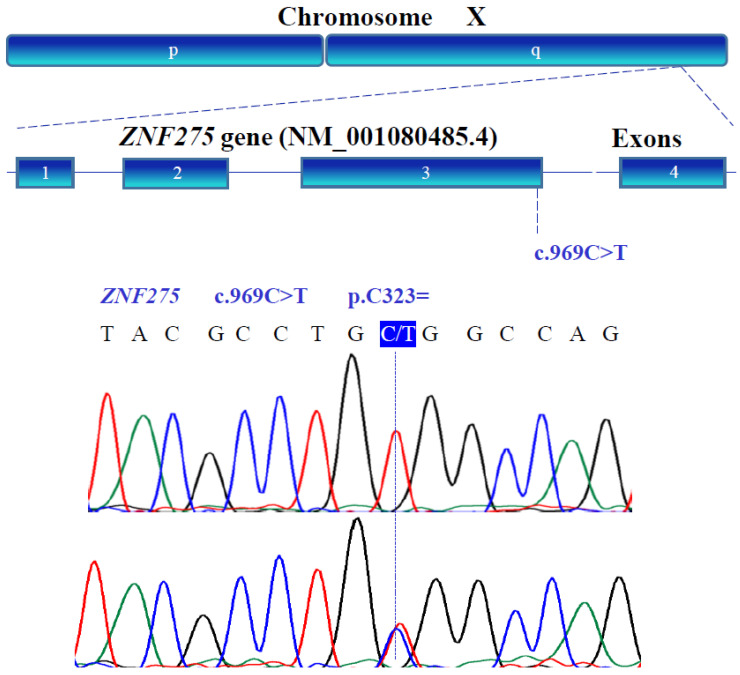
The *ZNF275* (NM_001080485.4) gene variant on the long arm of the X chromosome was verified by Sanger sequencing. A homozygous variant (TGT) and heterozygous variant (TGT/TGC) were found in exon 3 (blue box).

**Figure 8:**
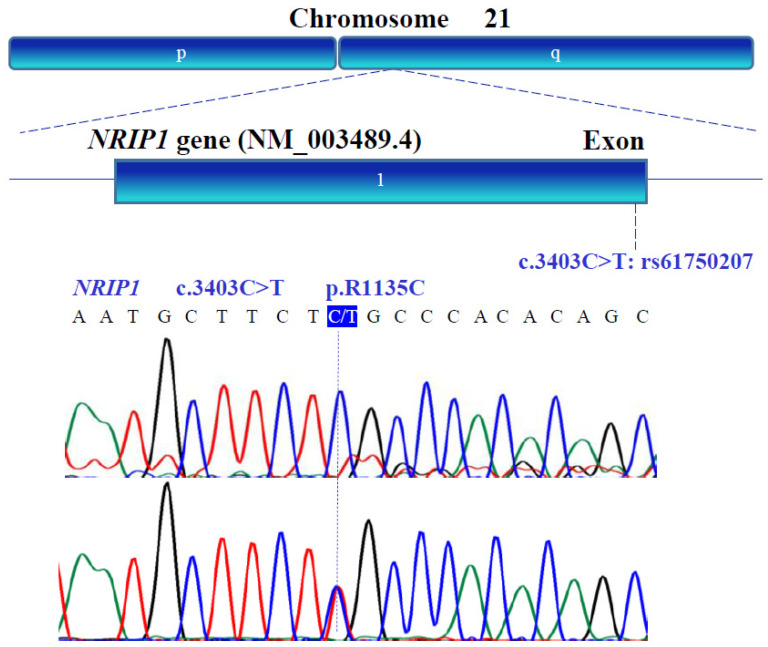
Exonic region of the human *NRIP1* (NM_003489.4) gene located on the long arm of chromosome 21. Nucleotide sequence analysis identified a polymorphic variant located in the end region of the intronless *NRIP1* gene (blue box).

The genetic variants of *WT1* (NM_024426.6), *NR0B1* (NM_000475.5), *NR5A1* (NM_004959.5), *LHX9* (NM_020204.3), *ZNF275* (NM_001080485.4), and *NRIP1* (NM_003489.4) were determined with bidirectional Sanger sequencing. Figure 3, Figure 4, Figure 5, Figure 6, Figure 7 and Figure 8 illustrate the molecular analysis in patients with POI and healthy subjects. Figure 3 depicts the three genetic variants in exon 1b of the *WT1* gene: GG, TT, and GT at position 213 in the cDNA, which encodes for a synonymous variant Pro-71 (NM_024426.6:c.213G>T; p.P71=); two variants in exon 1e: CC and TC at position 609 in the cDNA, which encodes for a synonymous variant Asn-203 (NM_024426.6:c.609T>C; p.N203=); two variants in exon 3: GG and GA at position 873 in the cDNA, which encodes for a synonymous variant Arg-291 (NM_024426.6:c.873A>G; p.R291=); three variants in exon 7: GG, AA, and GA at position 1122 in the cDNA, which encodes for a synonymous variant Arg-374 (NM_024426.6:c.1122 G>A; p.R374=). In Figure 4, we identified one non-synonymous and one synonymous variant in the *NR0B1* gene. There were two genotypic variants in exon 1b: CC and CT at position 353 in the cDNA, which encodes a non-synonymous variant Ala118Val (NM_000475.5: c.353C>T; p.A118V); three variants in exon 1c: AA, GG, and GA at position 498, which encodes for a synonymous variant Arg-166 (NM_000475.5: c.498G>A; p.R166=). The variant sequencing results for the *NR5A1* gene are shown in Figure 5. In exon 4b, two GG and GC variants were identified at position 437 in the cDNA, which encode for a non-synonymous change Gly146Ala (NM_004959.5: c.437G>C; p.G146A). A non-coding genotype (CC, TT, and CT) was localized to IVS4−20 (NM_004959.5: IVS4−20C>T). The automated sequencing in Figure 6 depicts two different GG and GC non-coding changes in intron 2 (NM_020204.3: IVS2−12G>C) and other non-coding genotypes IVS3+13 CC, TT, and CT (NM_020204.3:IVS3+13C>T) of the *LHX9* gene; in addition, in exon 4, there were two different TT and TC genotypes at position 741 in the cDNA, which encode for a synonymous variant Asn-247 (NM_020204.3: c.741T>C; p.N247=). Figure 7 illustrates the two different TT and CT genotypes at position 969 in the cDNA, which encodes for a synonymous variant Cys-323 (NM_001080485.4: c.969C>T; p.C323=) in exon 3 of the *ZNF275* gene, and Figure 8 describes the two different CC and CT genotypes at position 3403 in the cDNA, which encodes for a non-synonymous variant Arg1135Cys (NM_003489.4: c.3403C>T; p.R1135C) of the *NRIP1* gene.

### 3.4. Genotype Distribution

The study participants were genotyped using SSCP, and all of the variations were confirmed to be present in 100 healthy subjects to determine heterozygote and homozygote carrier state. Table 1 shows the genotyping data and allele frequencies. All of these data showed a lack of statistically significant difference between the alleles of the six candidate genes with genotypic variants in the study population with POI and the healthy subjects (*p* > 0.05). In both groups, the genotypes and allelic variants for *WT1*, *NR0B1*, *NR5A1*, *LHX9*, *ZNF275*, and *NRIP1* genes were in H-W equilibrium. Therefore, the genotypic or allelic variants found in the *WT1*, *NR0B1*, *NR5A1*, *LHX9*, *ZNF275*, and *NRIP1* genes were not associated with POI in this study population.

### 3.5. Pathogenic Analysis

Table 2 illustrates the prediction scores for the genetic variants identified from study patients with POI and healthy subjects. Aside from the deleterious association indicated by PROVEAN and VarSite for the *NRIP1* gene, all four programs (PolyPhen-2, PROVEAN, MutationTaster, and VarSite) used to predict the impact of genetic alternations on protein function indicated only neutral or benign effects; therefore, none of the genetic substitutions reported in this study were disease-associated variants.

## 4. Discussion

The present study revealed 13 single-nucleotide variants (SNVs), and to the best of our knowledge, seven of them are reported for the first time (*WT1* (c.609T>C, c.873A>G, c.1122G>A), *NR0B1* (c.353C>T, c.425G>A), *NR5A1* (c.437G>C: rs1110061, IVS4-20C>T), *LHX9* (IVS2-12G>C: rs12122995, IVS3+13C>T: rs13376365, c.741T>C: rs12046958), *ZNF275* (c.969C>T), and *NRIP1* (c.3403C>T: rs61750207)). Five were novel synonymous SNVs (sSNVs), one was a novel non-synonymous SNV (nsSNV), and one a novel non-coding SNV (ncSNV) (Table 1); these sSNVs were not found in the catalogue of common human genetic variations (1000 Genomes Project: https://www.ncbi.nlm.nih.gov/variation/tools/1000genomes/, accessed on 1 March 2022 and Genome Aggregation Database: https://gnomad.broadinstitute.org/, accessed on 1 March 2022).

Theoretical and experimental evidence supports the non-neutrality of synonymous alleles or sSNVs in animals and the human population [35,36]. Examples of functionality associated with synonymous sites include maximized translational efficiency, optimized mRNA stability, and efficient splicing control. In pigs, the c.258G>A synonymous mutation alters *IGF1* gene expression and affects IGF1 folding and its interactions with the IGF1R [37]. Kirchner et al. [38] reported that the c.2562T>G; p.T854 = sSNV induced local changes in translation velocity, giving rise to more stable channels with a greatly reduced single-channel conductance. A synonymous coding variant c.1437G>C/p.Arg479 = was reported in patients with X-linked sideroblastic anemia (XLSA), where the *ALAS2* mRNA transcribed from the c.1437C allele is spliced less efficiently and/or degraded via nonsense-mediated decay [39]. A synonymous coding base change was identified in the mRNA splicing site of the *CYP21A2* gene. The novel pathogenic variant c.1116C>T; p.Ser372 = is associated with congenital adrenal hyperplasia (CAH) due to 21-hydroxylase deficiency [40]. Moreover, a pathogenic synonymous variant p.Ser324 = (c.972G>A) in the *SLC2A1* gene is associated with paroxysmal exercise-induced dyskinesia [41]. Despite these findings, no relationship was found between the seven genotypic synonymous variants identified in this study (sSNVs in the *WT1* ((c.213G>T; p.P71=), (c.609T>C; p.N203=), (c.873A>G; p.R291=), and (c.1122 G>A; p.R374=)), *NR0B1* (c.498G>A; p.R166=), and *ZNF275* (c.969C>T; p.C323=) genes; sSNV reported in the *LHX9* (c.741T>C; p.N247=) only reported in 1000 Genomes Project and Genome Aggregation Database) and POI; rather, these findings suggest that the identified synonymous genotypes are more likely involved in the genomic variability of the Mexican population. Future studies should attempt to determine the possible impact of these synonymous variants on mRNA splicing, translation, or stability using software and tools such as ESEFinder or MMSplice.

Genomic analysis identified three nsSNVs in *NR0B1* (c.353C>T; p.A118V), *NR5A1* (c.437G>C; p.G146A), and *NRIP1* (c.3403C>T; p.R1135C; only reported in the 1000 Genomes Project and Genome Aggregation Database) genes from patients with POI. It is suggested that the novel coding variant p.A118V in *NR0B1* gene identified from patients with POI and healthy subjects could only be enriching the genetic diversity; however, future functional studies should examine the role of non-coding variants in RNA biogenesis. Nevertheless, similar to the allelic substitution c.437G>C in the *NR5A1* gene not correlating with POI in the present study, this genotypic variant did not affect the risk of hypospadias in male Caucasian patients [42], nor was it associated with congenital lipoid adrenal hyperplasia [43]; therefore, this genotypic variant does not confer the risk of developing a genetic disease, but rather represents a component contributing to the variability of the human genome.

Three ncSNVs were identified from patients with POI and healthy subjects. In this study, the novel genotypic variant (IVS4−20C>T) in the *NR5A1* gene was not found to contribute to the risk of developing POI and, to date, might only represent an allelic frequency for comparative genomic analysis. Although the molecular assays identified an association between non-coding genotypes and the predisposition for and clinical outcome of genetic disorders, the underlying mechanisms of many variants are still unclear [44,45]. Therefore, future experimental assays are warranted on the role non-coding variants play in cell function. The two non-coding allelic variants (IVS2−12G>C and IVS3+13C>T) of the *LHX9* gene have not been published, and the SNVs were only reported in the 1000 Genomes Project and Genome Aggregation Database.

In this study, the genomic data do not support a correlation between the identified variants and POI. The functional significance of these (*WT1* (c.213G>T, c.609T>C, c.873A>G, c.1122G>A), *NR0B1* (c.353C>T, c.425G>A), *NR5A1* (c.437G>C, IVS4-20C>T), *LHX9* (IVS2-12G>C, IVS3+13C>T, c.741T>C), *ZNF275* (c.969C>T), and *NRIP1* (c.3403C>T)) variants is unknown, and future studies are needed to establish the biological mechanisms that regulate these allelic variants, in addition to a detailed functional characterization of each allelic variant. These seven novel and three previously reported (*AKR1C2* (c.666T>C; p.H222=) and *AKR1C3* (c.538T>C; p.P180S and c.596G>A; p.R199Q)) genomic variants [46] likely contribute to genome sequence variability in the Mexican population. Given that the genetic mechanisms regulating female reproduction and the impact of non-genetic factors remain largely unclear, genotypic insight from this and future studies are essential for developing effective assays and treatments to optimize female reproductive function, preserve fertility, and improve the quality of life of women with POI.

## 5. Conclusions

Here, 13 genotypic variants or SNVs were identified in the *WT1*, *NR0B1*, *NR5A1*, *LHX9*, *ZNF275*, and *NRIP1* genes that lack any association with POI; similarly, no nucleotide variations were detected in the *ZFP92* and *INSL3* genes. Since all of these allelic variants were also found in POI-free subjects, none were specific to this clinical disorder. Seven of the allelic variants are novel SNVs, five are unpublished SNVs only mentioned in the 1000 Genomes Project and Genome Aggregation Database, and only one variant (c.437G>C; p.G146A) has been published. Based on these findings, the genetic variants identified in this study are likely components underlying the variability of the human genome rather than this complex disease.

## Figures and Tables

**Table 1:** A summary of the genotype and allele frequencies for the *WT1* (NM_024426.6), *NR0B1* (NM_000475.5), *NR5A1* (NM_004959.5), *LHX9* (NM_020204.3), *ZNF275* (NM_001080485.4), and *NRIP1* (NM_003489.4) genes from patients with POI (n = 11) and healthy controls (n = 100) in the Mexican population.

Gene	Nucleotide Variants	Genotype	Reference SNP	Function	Genotype Frequencies (POI)	Allele Frequencies (POI)	Genotype Frequencies (Controls)	Allele Frequencies (Controls)
*WT1*	c.213G>T	G/G	rs2234582	Synonymous	0.09	p = 0.135	0.1	p = 0.15
		T/T			0.82	q = 0.865	0.8	q = 0.85
		G/T			0.09		0.1	
	c.609T>C	C/C	This study	Synonymous	0.91	p = 0.955	0.93	p = 0.965
		T/T			0.0	q = 0.045	0.0	q = 0.035
		T/C			0.09		0.07	
	c.873A>G	G/G	This study	Synonymous	0.91	p = 0.955	0.9	p = 0.95
		A/A			0.0	q = 0.045	0.0	q = 0.05
		A/G			0.09		0.1	
	c.1122G>A	G/G	This study	Synonymous	0.18	p = 0.41	0.13	p = 0.39
		A/A			0.36	q = 0.59	0.35	q = 0.61
		G/A			0.46		0.52	
*NR0B1*	c.353C>T	C/C	This study	Non-Synonymous	0.91	p = 0.955	0.94	p = 0.97
		T/T			0.0	q = 0.045	0.0	q = 0.03
		C/T			0.09		0.06	
	c.425G>A	A/A	This study	Synonymous	0.18	p = 0.36	0.16	p = 0.355
		G/G			0.46	q = 0.64	0.45	q = 0.645
		G/A			0.36		0.39	
*NR5A1*	c.437G>C	G/G	rs1110061	Non-Synonymous	0.82	p = 0.91	0.90	p = 0.95
		C/C			0.0	q = 0.09	0.0	q = 0.05
		G/C			0.18		0.1	
	IVS4−20C>T	C/C	This study	Intronic	0.09	p = 0.225	0.08	p = 0.255
		T/T			0.64	q = 0.775	0.57	q = 0.745
		C/T			0.27		0.35	
*LHX9*	IVS2−12G>C	G/G	rs12122995	Intronic	1	p = 1	0.9	p = 0.95
		C/C			0.0	q = 0	0.0	q = 0.05
		G/C			0.0		0.1	
	IVS3+13C>T	C/C	rs13376365	Intronic	0.09	p = 0.365	0.13	p = 0.39
		T/T			0.36	q = 0.635	0.35	q = 0.61
		C/T			0.55		0.52	
	c.741T>C	T/T	rs12046958	Synonymous	0.09	p = 0.545	0.05	p = 0.525
		C/C			0.0	q = 0.455	0.0	q = 0.475
		T/C			0.91		0.95	
*ZNF275*	c.969C>T	T/T	This study	Synonymous	0.82	p = 0.91	0.92	p = 0.92
		C/C			0.0	q = 0.09	0.0	q = 0.08
		C/T			0.18		0.08	
*NRIP1*	c.3403C>T	C/C	rs61750207	Non-Synonymous	0.91	p = 0.955	0.98	p = 0.99
		T/T			0.0	q = 0.045	0.0	q = 0.01
		C/T			0.09		0.02	

**Table 2:** A summary of the predicted functional effects of the *WT1* (NM_024426.6), *NR0B1* (NM_000475.5), *NR5A1* (NM_004959.5), *LHX9* (NM_020204.3), *ZNF275* (NM_001080485.4), and *NRIP1* (NM_003489.4) variants from patients with POI and healthy controls. PolyPhen-2 (benign with a score of 0.0, possibly damaging with a score of 0.5, or probably damaging with a score of 1.0), PROVEAN (neutral effect for scores above the threshold or deleterious effect for scores equal to or below the predefined threshold), MutationTaster (scores range from 0.0 to 215 but no values provided for amino acid insertions/deletions), and VarSite (scored according to Fauchère and Pliska’s hydrophobicity scale).

Gene	Location	Nucleotide Substitution	Amino Acid Substitution	PolyPhen-2	PROVEAN	MutationTaster	VarSite
*WT1*	Exon 1	c.213G>T	p.P71=	ND	Neutral (0.000)	ND	ND
	Exon 1	c.609T>C	p.N203=	ND	Neutral (0.000)	Benign (0.000)	Benign (0.0)
	Exon 3	c.873A>G	p.R291=	ND	Neutral (0.000)	Benign (0.000)	Benign (1.29)
	Exon 7	c.1122G>A	p.R374=	ND	Neutral (0.000)	Benign (0.000)	Benign (0.32)
*NR0B1*	Exon 1	c.353C>T	p.A118V	Benign (0.041)	Neutral (−0.194)	Benign (64)	Benign (1.37)
	Exon 1	c.498G>A	p.R166=	ND	Neutral (0.000)	Benign (0.000)	Benign (0.61)
*NR5A1*	Exon 4	c.437G>C	p.G146A	Benign (0.000)	Neutral (1.516)	Benign (60)	Benign (0.56)
	Intron IV	IVS4−20C>T	ND	ND	ND	ND	ND
*LHX9*	Intron II	IVS2−12G>C	ND	ND	ND	ND	ND
	Intron III	IVS3+13C>T	ND	ND	ND	ND	ND
	Exon 4	c.741T>C	p.N247=	ND	Neutral (0.000)	Benign (0.000)	Benign (0.74)
*ZNF275*	Exon 3	c.969C>T	p.C323=	ND	Neutral (0.000)	Benign (0.000)	Benign (−0.22)
*NRIP1*	Exon 1	c.3403C>T	p.R1135C	Benign(0.003)	Deleterious (−2.796)	Benign (180)	Deleterious (2.55)

ND = Not determined; scores are shown in parentheses.

## Data Availability

Not applicable.

## Supplementary Materials

The following supporting information can be downloaded at: https://www.mdpi.com/article/10.3390/genes13040611/s1, Table S1: Oligonucleotide sequences used for PCR-[α^32^P]dCTP of the human *WT1* gene, Table S2: Oligonucleotide sequences used for PCR-[α^32^P]dCTP of the human *NR0B1* gene, Table S3: Oligonucleotide sequences used for PCR-[α^32^P]dCTP of the human *NR5A1* gene, Table S4: Oligonucleotide sequences used for PCR-[α^32^P]dCTP of the human *LHX9* gene, Table S5: Oligonucleotide sequences used for PCR-[α^32^P]dCTP of the human *ZFP92* gene, Table S6: Oligonucleotide sequences used for PCR-[α^32^P]dCTP of the human *ZNF275* gene, Table S7: Oligonucleotide sequences used for PCR-[α^32^P]dCTP of the human *INSL3* gene, Table S8: Oligonucleotide sequences used for PCR-[α^32^P]dCTP of the human *NRIP1* gene.
