# Supplementary material for: WT1, NR0B1, NR5A1, LHX9, ZFP92, ZNF275, INSL3, and NRIP1 Genetic Variants in Patients with Premature Ovarian Insufficiency in a Mexican Cohort
Source: Genes (Basel). 2022 Mar 29;13(4):611. doi: 10.3390/genes13040611 (PMC9025227; doi:10.3390/genes13040611)
Supplement: Supplementary file 1 [file genes-13-00611-s001.zip › genes-1577624-supplementary.pdf]

**Table S1.** Oligonucleotide sequences used for PCR-[α-$^{32}$P]dCTP of the human *WT1* gene.

| Gene | Exon | Primer sequence 5´-3´ | Amplicon size in base pairs | Annealing temperature |
|---|---|---|---|---|
| ***WT1*** | 1a | cctactcattcacccaccca<br>ctaacttggcccagatgcc | 226 | 66°C |
| | 1b | tgcctacagcagccagag<br>tcacaggcagggcacag | 220 | 60°C |
| | 1c | tctgagccgcagcaaat<br>tcctgtttgatgaaggagtgag | 239 | 60°C |
| | 1d | gccggtgctggactttg<br>cagtgaactggccggaaa | 200 | 60°C |
| | 1e | cctgagcgccttcactg<br>gtgtcctagagcggagagt | 247 | 60°C |
| | 2 | cgctgacactgtgcttctctc<br>gactccacttggttccgctc | 184 | 60°C |
| | 3 | gcactcgctcagctgtcttc<br>ccaaggacccagacgcaga | 173 | 60°C |
| | 4 | aaactccattgcttttgaagaaac<br>cctttgaaatggttcaaacaggt | 222 | 60°C |
| | 5 | tttcactggattctgggatctg<br>tacccacgtcagtcctaact | 226 | 60°C |
| | 6 | cctgattgcagataagcatttcc<br>taagtaggaagaggcagtgcg | 228 | 60°C |
| | 7 | gcttaaagcctcccttcctctt<br>gaaaaggagctcttgaaccatgt | 242 | 60°C |
| | 8 | gcctttaatgagatccccttttcc<br>aatcaaccctagcccaagggaac | 208 | 60°C |
| | 9 | ctcactgtgcccacattgttag<br>ccctctcatcacaatttcattcca | 221 | 60°C |
| | 10 | cctgtctctttgttgcaagtgtc<br>acttgaaagcagttcacacactg | 231 | 60°C |

**Table S2.** Oligonucleotide sequences used for PCR-[α-$^{32}$P]dCTP of the human *NR0B1* gene.

| Gene | Exon | Primer sequence 5´-3´ | Amplicon size in base pairs | Annealing temperature |
|---|---|---|---|---|
| ***NR0B1*** | 1a | agaactgggctacgggc<br>tgccgtgggtggtcttta | 254 | 58°C |
| | 1b | aacgtggcgctcctgta<br>gggtggtcttcaccacaaa | 245 | 62°C |
| | 1c | cccgtggcactcctgtacc<br>tgctgcgggtggtcttc | 254 | 64°C |
| | 1d | ggtaaagaggcgctacca<br>ctggaagcagggcaagta | 282 | 58°C |
| | 1e | tcagcgggcctgttgaag<br>cgcctggtggtgaggat | 200 | 58°C |
| | 1f | tcggagcccagcatgct<br>ggcgcctaaggccagta | 259 | 58°C |
| | 2a | ctagcaaaggactctgtggtg<br>agttcgatgaatctgtcatggg | 175 | 58°C |
| | 2b | ctcagtgaacacaccaggatg<br>ctcatggtgaactgcactact | 227 | 58°C |

**Table S3.** Oligonucleotide sequences used for PCR-[$\alpha$-$^{32}$P]dCTP of the human *NR5A1* gene.

| Gene | Exon | Primer sequence 5´-3´ | Amplicon size in base pairs | Annealing temperature |
|---|---|---|---|---|
| ***NR5A1*** | 2 | tccctgacccgctgtccct<br>ttgcagctctcacacgtgag | 210 | 60°C |
| | 3 | cacggtgcagaacaacaagcactac<br>aaggccaatggtactatcccct | 287 | 60°C |
| | 4a | tgtttggaaaggatctgtggtggg<br>cctgtctccagcttgaagccatt | 157 | 60°C |
| | 4b | ccctgaaacagcagaagaaggcaca<br>agggtagaggtagccagccagt | 201 | 60°C |
| | 4c | tgggccactgggcgacttt<br>tgcaggatgagctcaggcacgtt | 171 | 60°C |
| | 4d | aagtctgagtacccggagccttat<br>agatgaaggtctggtcggccatt | 197 | 60°C |
| | 4e | cctgcaggagcccaccaaaa<br>aaggatggccctatccaaaggaca | 179 | 60°C |
| | 5 | tagttgggtctcagtgggaggagaa<br>tgaatcctggaagtgcacagcg | 228 | 60°C |
| | 6 | acccacgtcctctgactgt<br>tgtctccacctctctgtcact | 189 | 60°C |
| | 7a | cgcgatggtgaccgagaacctccc<br>gggcatctcgttgcccaggtgct | 187 | 60°C |
| | 7b | gccctgagcatgcaggccaaggagt<br>aatgaaccatgcggagccagcggt | 150 | 60°C |

**Table S4.** Oligonucleotide sequences used for PCR-[$\alpha$-$^{32}$P]dCTP of the human *LHX9* gene.

| Gene | Exon | Primer sequence 5´-3´ | Amplicon size in base pairs | Annealing temperature |
|---|---|---|---|---|
| ***LHX9*** | 1 | ttcactcggatgagctgaaag<br>tcggcttggtttaacagagg | 275 | 60°C |
| | 2 | ctcagccttgcggtgtg<br>tactcgggcgtaggaagtg | 268 | 60°C |
| | 3a | gctctgccttgcttcaacta<br>tgcggtggatactctcctt | 264 | 60°C |
| | 3b | catgaaggacagcctggtgta<br>ggggaaggagctctacgaattt | 279 | 60°C |
| | 4 | gcagttgtttgttcactgttact<br>gggaaatgtatgcacagtcaatg | 271 | 60°C |
| | 5a | acatcggtaaagaaatcaattggg<br>gggattggtcaggtctgttaaa | 204 | 60°C |
| | 5b | ctcagcagacagcggagc<br>tccccaaatatcttgtgggttag | 277 | 60°C |

**Table S5.** Oligonucleotide sequences used for PCR-[α-$^{32}$P]dCTP of the human *ZFP92* gene.

| Gene | Exon | Primer sequence 5´-3´ | Amplicon size in base pairs | Annealing temperature |
|---|---|---|---|---|
| ***ZFP92*** | 1 | acagggggctctttgcatttc<br>cagcctaagctcagccca | 175 | 62°C |
| | 2 | gggttgaattcccctgtgg<br>gggtgtgtgtatgtcgtgga | 200 | 62°C |
| | 3 | ccctgccctcagccatagt<br>tctttttaccccgatggggagc | 199 | 62°C |
| | 4a | tctctgacttggcatgagggt<br>ctggcacaggtaccgcttct | 249 | 62°C |
| | 4b | gggcttggggcagagttc<br>gtggatgcgctggtgctc | 211 | 68°C |
| | 4c | cccgagtgcggcaagctg<br>ggagctgcggctgaaggc | 285 | 68°C |
| | 4d | gccctacgcgtgcccaga<br>ctggctgaggcccgaacg | 226 | 62°C |
| | 4e | ccaccagcgcagccacag<br>ctagcctccgcgccgtctc | 227 | 68°C |
| | 4f | aacctattcaagcaccaggcag<br>tcgacgtccccaggaagg | 230 | 64°C |

**Table S6.** Oligonucleotide sequences used for PCR-[α-$^{32}$P]dCTP of the human *ZNF275* gene.

| Gene | Exon | Primer sequence 5´-3´ | Amplicon size in base pairs | Annealing temperature |
|---|---|---|---|---|
| ***ZNF275*** | 1 | aatccaacagatggggcctta<br>gtcaaagccagcagacaccat | 128 | 60°C |
| | 2 | ctcatgtcatctctggctgttg<br>gtctcccccaatttcctcacat | 190 | 60°C |
| | 3a | ccttcatcctctgcagactacac<br>ggactttaagccgaaaggtgtc | 255 | 60°C |
| | 3b | tctgcccatagaacatcattttgc<br>cactcctcgcactcgaagg | 276 | 60°C |
| | 3c | gagaagagggagcagatggaga<br>ctcctggcgatccaggaaatc | 249 | 60°C |
| | 3d | cttcggaaaagcctttcgcct<br>cgccggtgcttggtgagc | 244 | 60°C |
| | 4 | ccatacgggtgtccccact<br>ctcatagggtttcagtccgctg | 261 | 60°C |

**Table S7.** Oligonucleotide sequences used for PCR-[α-$^{32}$P]dCTP of the human *INSL3* gene.

| Gene | Exon | Primer sequence 5´-3´ | Amplicon size in base pairs | Annealing temperature |
|---|---|---|---|---|
| ***INSL3*** | 1 | gcccaggcgcctataaa | 286 | 62°C |
| | | acctcggcttcccagag | | |
| | 2a | aaaccatcctcctgcctcag | 172 | 62°C |
| | | cccaacactttggaaggtcaag | | |
| | 2b | ttcgtagagagggagtctca | 127 | 60°C |
| | | tttaaagctggacacagtga | | |
| | 3 | gtagcatgtcctctgtcgtttc | 271 | 60°C |
| | | acccaaggaggaatcagtagg | | |

**Table S8.** Oligonucleotide sequences used for PCR-[α-$^{32}$P]dCTP of the human *NRIP1* gene.

| Gene | Exon | Primer sequence 5´-3´ | Amplicon size in base pairs | Annealing temperature |
|---|---|---|---|---|
| ***NRIP1*** | 1A | catcgcactcaccacagaa | 244 | 60°C |
| | | tgcactgccagaaatgttaaag | | |
| | 1B | ctgggcataatgaagaggatcag | 290 | 60°C |
| | | ctgaagcaaagaggccagtaa | | |
| | 1C | tggttgacagtgtgcctaaa | 264 | 60°C |
| | | tcacatcaggaagattcgtatcag | | |
| | 1D | gtggagaaggatttaaggtgctat | 275 | 60°C |
| | | gcaacactaggtttaggtgagg | | |
| | 1E | gcaagattacaggctgttgc | 239 | 60°C |
| | | agctggtaactgccaacat | | |
| | 1F | gctatggccagattgcaagaa | 241 | 60°C |
| | | gcaaactattgttagcagcttgt | | |
| | 1G | aggttataagaactcactggaaaga | 242 | 60°C |
| | | tgggaacacagttggaataaga | | |
| | 1H | tgacagcagtggtgatgaaa | 242 | 60°C |
| | | ggccaagtagcaattgaagaag | | |
| | 1I | agctaaactcacaccagaaagt | 261 | 60°C |
| | | tccatttgatgaccagagagtg | | |
| | 1J | cagggtctcccatcaatctc | 262 | 60°C |
| | | ctctgctcttccactgacat | | |
| | 1K | tttagcacaatgtggaatgcag | 248 | 60°C |
| | | gcaactggaggacagtacg | | |
| | 1L | gggctttctggttctgaaata | 243 | 60°C |
| | | cttagcatcatggctcaagtg | | |
| | 1M | gccttgtgatgacttacaaattcc | 246 | 60°C |
| | | ctgtgactcctgtctgaatcatc | | |
| | 1N | gtctgctaagtcgattgctaaga | 259 | 60°C |
| | | ctaaatttcagctcttcctggttaag | | |
| | 1O | ccagaagtactgtatgggtcct | 273 | 60°C |
| | | gctaaattcaggtttgctgttgg | | |
| | 1P | gtaactctgtggctgacagtaaa | 273 | 60°C |
| | | cccacttaatgggtcctttctc | | |
| | 1Q | tgaatgggtgttccatgcc | 260 | 60°C |
| | | agctttcgtttctgcagtagg | | |
| | 1R | gtcacagccaaagaagagtta | 236 | 60°C |
| | | acagatctcaagttcatactca | | |
